# The Largest Electron Differential Energy Flux Observed at Mars by the Mars Express Spacecraft, 2004–2016

**DOI:** 10.1029/2018JA025311

**Published:** 2018-08-22

**Authors:** R. A. Frahm, J. D. Winningham, A. J. Coates, J.‐C. Gérard, M. Holmström, S. Barabash

**Affiliations:** ^1^ Southwest Research Institute San Antonio TX USA; ^2^ Mullard Space Science Laboratory University College London Surrey UK; ^3^ Laboratory of Planetary and Atmospheric Physics Université de Liège Liège Belgium; ^4^ Swedish Institute of Space Physics Kiruna Sweden

## Abstract

The goal of this paper is to understand the processes by which solar wind electrons are energized in the Martian magnetosphere and how this compares to processes at Venus and Earth. Each is unique in the source of its magnetic field topology and how this influences electron energization. To achieve this goal, 24 million spectra spanning 13 years have been examined using the electron spectrometer from the Mars Express spacecraft between about 12,000 km and about 250 km altitude, and from all latitudes and local times. The top 10 largest differential energy flux at energies above the differential energy flux peak have been found: seven spectra from the magnetosheath near noon, three from the dark tail (the largest two from the middle and ionospheric edge of the magnetosheath). Spectral comparisons show a decade range in the peak of the electron distributions; however, all distributions show a similar energy maximum dictated by solar wind/planet interaction. Similarly derived, the largest Venus spectrum occurred near the magnetosheath bow shock and had the same shape as the most intense Mars inner magnetosheath spectrum. The Mars and Venus dayside spectra compared to the Mars nightside spectrum that included an enhanced optical signal attributed to discrete “auroral” precipitation show a similar shape. These spectra are also compared to a selected auroral zone electron spectra from the Earth. The Mars and Venus results suggest that there is no more energy needed to generate electrons forming the nightside precipitation than is gained during the solar wind/planet interaction.

## Key Points

- Top 10 orbit‐selected electron energy spectra from Mars are identified from MEx ASPERA‐3 ELS between 2004 and 2016
- The Mars and Venus dayside electron spectra are similar, and a discrete “auroral” spectrum from the nightside tail of Mars is also similar
- No more energy than that supplied by the solar wind‐planet interaction is required to produce discrete auroral spectrum from the nightside tail of Mars

## 1 Introduction

The goal of this paper is to understand the processes by which solar wind electrons are energized in the Martian magnetosphere and how this compares to processes at Venus and Earth. Each planet is unique in the source of its magnetic field topology and how this influences electron energization. To achieve this goal, 24 million spectra spanning 13 years with the Mars Express (MEx) spacecraft (Chicarro et al., 2004) have been examined. MEx has been making measurements at Mars for over a solar cycle. During this time, MEx has visited nearly all local times from altitudes as high as 12,000 km to as low as 250 km.

The spectrum of electrons in the 1 eV to 20 keV energy range in the Mars magnetosphere measured by the MEx spacecraft has the characteristic that the number of electrons decreases with increasing energy, typically showing a change in the slope with an increase in energy. This slope change can be variable, with the exact energy location of where the slope changes, the number of slope changes, and magnitude of the slopes variable as well. In some cases, the number of electrons may increase to form a localized maximum, indicating a region of larger intensity. If this same spectrum is expressed in terms of the differential energy flux (DEF), the amount of energy carried by these electrons is revealed: Generally, at low energies, there are many electrons that do not carry very much energy, so their DEF is low, and when the energy is very high, each electron carries more energy, but there are fewer electrons resulting again in a low DEF. The electron spectrum in the magnetosphere of Mars can be qualified by two factors: the DEF of the electrons and the energy of the electrons. Those electron energy spectra maximized along these two factors will describe the maximum extent of the shape of the electron spectrum. The DEF is typically its highest somewhere between very low energy (1 eV) and very high energy (20 keV), and the exact location is dependent on where the electron spectrum is measured. If the electrons enter into an area that is collisionally dominated, the higher‐energy electrons will penetrate deeper into the medium than lower‐energy electrons. Unlike at the Earth, at Mars and Venus the magnetic field (which directs charged particles) can be significantly horizontal, as draped field lines penetrate the atmosphere. This magnetic field configuration leads to energy deposited horizontally instead of vertically as at the Earth. Currently, there is no Tsyganenko (2002a, 2002b) style magnetic field model available for Mars and Venus that would allow for prediction of where energy is deposited using the continuous slowing‐down approximation (Sharber et al., 1996).

We approach this study without preconceived notions of the expected shape of the electron spectrum or from where the population of the spectrum originates. Our goal is to find the largest electron spectrum at energies above the DEF peak observed in the Mars environment over the time period of a solar cycle in order to determine the energized electron spectrum. To our knowledge, this type of study has never before been performed. Beyond the scope of this study is to evaluate through models the effect depositing these electrons on the atmosphere on Mars; this is left as an exercise for the future.

Energetic electron spectra at Mars have been presented (Brain et al., 2006; Lundin et al., 2006), which can contain enough energy to excite an optical signature when they reach the Martian atmosphere (Bertaux et al., 2005; Leblanc et al., 2006). However, can it be qualified as to how large are these spectra relative to what is uncovered by this study? Are the processes required to generate precipitating spectra comparable, and will this study uncover them as being large and dominant around the planet? At the Earth in the discrete auroral zone, spectra resembling that found at Mars have been observed. Just how do discrete auroral zone spectra at the Earth compare to the spectra that are uncovered with this study of observed electron spectra at Mars? In this paper we restrict this comparison of the largest electron spectra that we find to those that generate the discrete optical emission on Mars as opposed to other types of Martian optical emission (diffuse as in Schneider et al., 2015, or the dayside proton as observed by Ritter et al., 2018).

For this paper we use the MEx data collected through 2016 to determine the largest electron spectra observed at Mars, without pretense as to whether or not the electron spectrum indicates acceleration by various processes. We compare a large spectrum found at Mars with the largest spectrum found at Venus. One of the largest spectra found at Mars has been identified as containing the time when an enhanced optical signature has been identified as “aurorally” produced (see Gérard et al., 2015, Table 1), and this has been included in the comparison. Finally, these spectra at Mars and Venus are compared to one from the Earth's auroral zone.

## 2 Instrumentation

The Analyzer of Space Plasmas and Energetic Atoms (ASPERA‐3) experiment (Barabash et al., 2006; Barbash et al., 2004) contains an electron spectrometer (ELS). The ELS is a spherical top hat sensor with a 4° by 360° angular width separated into 16 sectors, each 22.5° wide. It contains two linear power supplies that produce different potentials across its electrostatic deflection plates that set the acceptance energy. Each power supply produces 4,096 voltage values between 0 and 20.99 V (to measure a maximum of about 150 eV in energy) in its low range and in its high range, 0 to 2,800 V (to measure a maximum of about 20 keV in energy). During the period of time between launch in 2003 and operation in 2016 (the time range analyzed in this paper), ELS has operated in several modes. ELS is fully programmable, so it could continue to operate in these modes or new modes not yet defined as the instrument continues to acquire new electron spectra. The various modes used to produce data to date are as follows: (1) at high time resolution (32 samples per second at a fixed energy); (2) linear stepping‐sampled energy between 1 and 127 eV with a 4‐s time resolution and 1‐eV energy resolution; (3) a 32‐point log‐sampled energy spectrum with a 1‐s time resolution from about 9 to about 150 eV; (4) a 127 log‐sampled energy spectrum between about 0.5 eV and about 20 keV with about an 8% energy resolution every 4 s; and (5) beginning in 2014, the 127 log‐sampled energy spectrum with reduced time resolution (approximately localized to the solar wind) of 8, 16, or 32 s.

Energy spectra were corrected for instrument backgrounds, which are composed of any source of signal that exhibits an energy‐independent behavior. Examples of energy‐independent noise are thermally generated noise within the microchannel plate sensor or electronics, penetrating radiation through the sides of ELS from MeV ions, cosmic ray noise within the microchannel plate, and so on. Instrument backgrounds for each ELS sector were determined by accumulating the instrument counts when ELS measured large electron energies, that is, above 10 keV. This was approximately 10 energy steps. For the data examined in this paper, background values for each sector were accumulated every 5 min and then averaged, generating a sector‐dependent background level. Background values were subtracted from the science signal that produced some negative flux values. For the purposes of this research, these negative values were kept when generating averages within energy ranges and discarded when examining the largest fluxes within the energy ranges. The energy of 10 keV was chosen because at the sensitivity level of the instrument, environmental counts rarely extend into the 10 keV energy range and most of the counts observed above 10 keV are due to energy‐independent sources such as penetrating radiation, thermal noise, or instrument noise.

The ELS instrument is mounted to the main unit of the ASPERA‐3 experiment so that the ELS symmetry axis is perpendicular to the ASPERA‐3 main unit. The main unit is mounted on a rotatable scan platform attached to the upper deck of the spacecraft (+*Z*), so the scanner rotation axis is about the spacecraft *Z* direction. ELS sector pairs 0 and 15 view along either side of the −*Z* spacecraft direction, and pairs 7 and 8 view along either side of the +*Z* spacecraft direction. Thus, in 180° of scanner rotation, ELS has the capability of measuring the full 4π sr volume.

From launch until January of 2006, the ASPERA‐3 scanner was configured in its launch position (see Figure 5 of Barabash et al., 2006, for a picture of the ASPERA‐3 scanner hardware in its launch position, i.e., scanner rotation angle of 90°) such that the ELS sensor plane was parallel to the −*Y* side of the spacecraft and the ELS sector plane is parallel to the spacecraft *X*‐*Z* plane. After January of 2006, the scanner rotated with a limited number of scans, parking at different locations. However, the preferred parking location of the scanner (more than about 90% of the time) was at about 10° to the *X*‐*Z* spacecraft plane, which placed ELS measurements close to the *X*‐*Z* spacecraft plane. This position was preferred because the ASPERA‐3 main unit was thermally stabilized. In this orientation, ELS sectors 0, 12, 13, 14, and 15 look at the spacecraft, observing electrons emanating from the spacecraft surface, and are not directly measuring the space environment.

## 3 Time/Spatial Coverage

The MEx spacecraft orbital motion exhibits precession in local time, covering 24 hr of local time in about 2 years of Earth time. The MEx orbit has changed since its arrival at Mars in 2004. Apoapsis was lowered from an altitude of ~12,000 to just under 10,000 km and periapsis was raised from ~250 to ~350 km. In general, there are about three orbits of the MEx spacecraft around Mars each day.

The ASPERA‐3 experiment does not operate continuously. For the majority of time, ASPERA‐3 operated beginning 20 min prior to contact with the nominal bow shock location (Vignes et al., 2000) to 20 min after passing the nominal bow shock location, collecting measurements through periapsis around Mars. Even though there are about three orbits of MEx per day around Mars, MEx power and telemetry is limited; and this, for periods of time, has limited the operation of ASPERA‐3. About once per Earth year there is an eclipse season, when parts of MEx orbit are in the shadow of Mars. The power available is then limited, and this limits the science operations. The distance between Earth and Mars varies over time, and it is at a minimum (maximum) during oppositions (solar conjunctions). The rate at which data can be downlinked to Earth increases with decreasing distance between the planets and will vary over time. The availability of large receiver antennas at Earth will affect the data rate. During low data rate periods, science observations are limited to what can be downlinked. MEx does not operate during solar conjunctions, defined when observed from Earth, Mars passes behind the Sun and telemetry is blocked. During its first 13 years of coverage, the MEx spacecraft underwent several safeholds, which excluded the collection of measurements and other times when spacecraft power limited ASPERA‐3 use. With the arrival of the MAVEN (Jakosky et al., 2015) and Trace Gas Orbiter (Vandaele et al., 2015) missions, ASPERA‐3 extended its measurement coverage into the solar wind on a more regular basis by changing instrument modes to include reduced time resolution. This allowed ASPERA‐3 to make measurements over a larger portion of the MEx orbit while remaining within its telemetry allotment. All of these conditions lead to unequal time coverage; however, this still represents over 24 million ELS spectra taken within the Martian environment.

The MEx spacecraft does not carry a magnetometer. As such, plasma data from the MEx spacecraft cannot be oriented to the local magnetic field. This means that the precipitating or escaping particle distributions cannot uniquely be identified.

## 4 Sorting Methodology

The differential number flux (DNF) describes the number of electrons that crosses a plane of a certain size within an angular volume, and with respect to energy of the electron for a given amount of time. The DNF at Mars shows a large number of electrons at the smallest energies and a small number of electrons at high energy. Even though electrons are more abundant at low energy, they do not carry a significant amount of energy, whereas at high energy, each electron carries a substantial amount of energy, but there are relatively few electrons. The electron distribution function (DF) describes how the phase space density is distributed in energy or velocity. The DNF has a shallower slope than the DF, which indicates that the phase space density is concentrated at the lower energies. The DEF weights the electron distribution by the amount of energy contained in the electron spectrum. It is similar to the DNF but describes the amount of energy crossing a surface area within an angular volume and with respect to energy of the electron for a given amount of time. Thus, the DEF describes the amount of kinetic energy (energy flux) carried by the electrons at each given energy.

In order to examine the electron spectra from the 11‐year solar cycle, all ELS modes were examined between MEx arrival at Mars at the beginning of 2004 and the end of 2016. Four different energy range categories were examined in order to cover the energy range where the maximum DEF could be obtained: (1) ~150 to 500 eV, (2) 500 eV to 1 keV, (3) 1 to 5 keV, and (4) 5 to 10 keV. Data at the instrument time resolution was examined in two classes: (A) the largest single value of DEF and (B) the largest average DEF within each category. The classes were chosen to identify spectra that were very peaked and to distinguish these from those that had gradually changing shapes.

One orbit of data is examined at a time, and each ELS sector is handled independently. Comparisons are made directly for class A, saving the largest single DEF in each category (4 values × 16 sectors):

(1) $$\mathrm{DEF}\;\left(\text{category},k\right)=\mathrm{MAX}\;\left(\mathrm{DEF}\;\left(\text{category}\;\mathrm{low}\;\text{energy},k\right),\ldots ,\mathrm{DEF}\;\left(\text{category high energy},k\right)\right)$$

where *k* is the ELS sector number and comparisons are made over all sector energies within the category energy range. Class B averages are determined by comparing the straight average within each category (also 4 values × 16 sectors):

(2) $$N\;\left(\text{category},k\right)=\sum_{\text{category}\;\mathrm{low}\;\text{energy}}^{\text{category high energy}}1$$

where *N* is the number of samples in each category and ELS sector, and

(3) $$\mathrm{DEF}\;\left(\text{category},k\right)=\frac{1}{N\;\left(\text{category},k\right)}\sum_{i=\text{category}\;\mathrm{low}\;\text{energy}}^{\text{category high energy}}\mathrm{DEF}\;\left(i,k\right)$$

where *i* is an energy step index for energies within the category energy range. The averages described by equation (3) are to determine the maximum value in an orbit. The results from one orbit are compared to those from previous orbits. The final values from each orbit are independently compared to and ranked against the other orbital data. If the processed orbital values are within the top 10 under each class and category for each ELS sector, they are inserted into their proper place in rank order before the next orbit is processed. Saved are the time for each spectrum and their class/category DEF value for each ELS sector.

For each of the 16 ELS sectors, the times with the top 10 largest values were selected with the requirement that there be only one identification per sensor per category per class per orbit. Without imposing this requirement, it was discovered that often the top 10 spectra identified were all from the same spacecraft pass. This defeated the purpose of identifying the top 10 most intense time periods and not the top 10 spectra from the same time period. The search criteria resulted in 1,280 identified spectra (16 sectors × 4 categories × 2 classes × 10 time periods). These were examined to find only the top 10 that showed the largest flux at energies above the DEF peak (keV electrons from spectra with the largest DEF that are continuous up to keV energies).

**Figure 1:**
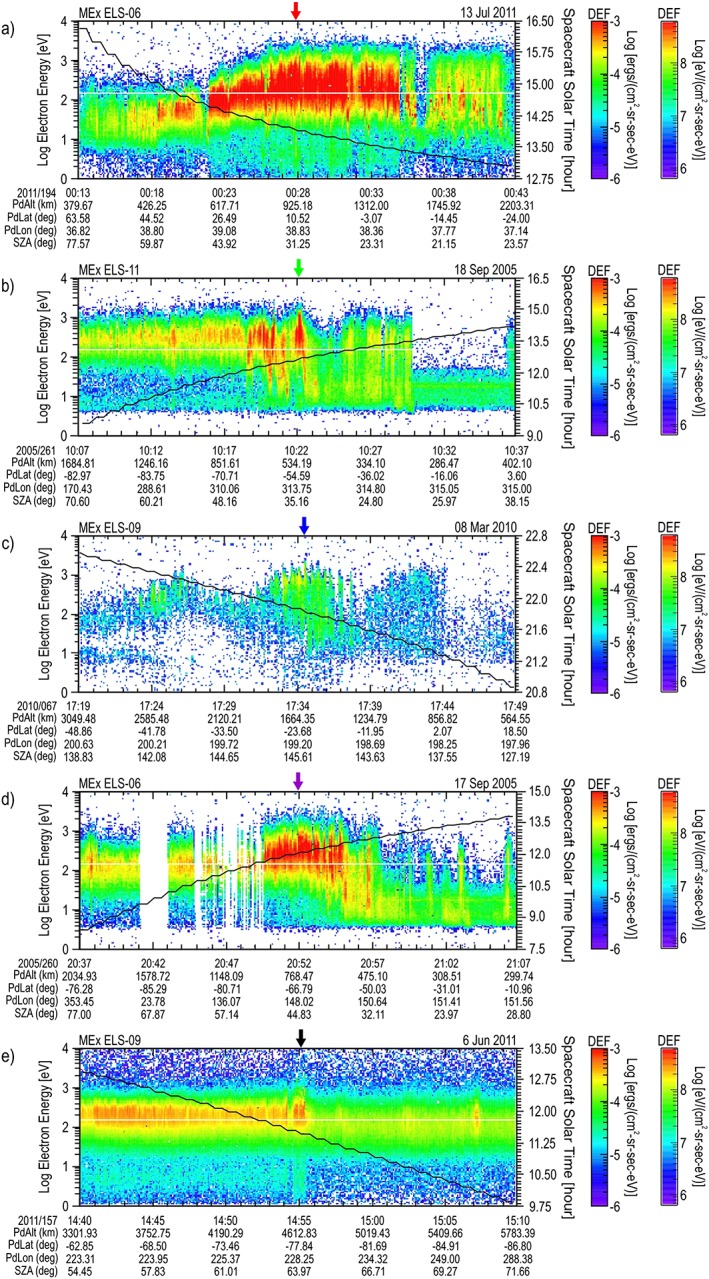
Top five maximum differential energy flux (DEF) energy‐time spectrograms. The order (a–e) is that listed in Table 1. For each spectrogram, an overlay shows the solar local time (hr) with the scale on the right axis. At the bottom of each plot are given the planetodetic altitude (PdAlt in km), latitude (PdLat in deg), and longitude (PdLon in deg) along with the solar zenith angle (SZA in deg) of the spacecraft. An arrow at the top of each panel marks the location of the largest DEF spectrum (the color of this arrow corresponds to the color of the orbit presented in Figure 3 and the maximum DEF spectrum presented in Figure 5).

During operation of ELS over such a long time period, several artifacts were observed. These artifacts included lone maximum high‐flux single points, times of instrument interference, power supply thermal disruptions, and other times of data noise from unknown spacecraft sources that occurred. Some of these artifacts occurred in one sector, while others occurred in many sectors. Lone maximum high values typically were noticed above the contiguous electron spectrum and represented a single point in a single sector. These were not reproducible and were random, high statistical variations in the Poisson count, which occurred during low count rates in any sector. Instrument interference is observed mostly restricted to sectors 0 and 15, shows high intensity fluxes that increase or decay in energy with time and repeat on minute time scales. This type of noise occurred mainly in 2005 and has not been observed since the science instrument function of MEx was separated from relay communications. Power supply thermal disruptions are observed mainly in 2005 when there started to be breakdown in the ELS power system. This first appeared as infrequent millisecond dropouts in 2004 and increased to minute time scales in 2006, it was discovered that the heat from the Sun was causing thermal breakdown. The ASPERA scanner was positioned to cool the ASPERA main unit, which reduced the temperature. Data occurring in 2006 after the ASPERA scanner was repositioned is unaffected by this artifact, but some data from 2005 show this effect (e.g., this artifact is observed in Figure 1d). Rarely, there have been times when the transition between spacecraft operational modes was not handled cleanly. This caused the data from the ELS to show high levels of counts over the high portion of the energy spectrum. Typically, all sectors would be affected the same, and this interference would last on the order of seconds. When searching the data for large spectra, events with artifacts are highlighted. Most events showing artifacts were trapped and were excluded from the search. They do not represent environmental spectra.

To emphasize, the shape of the spectrum is not predetermined. The sorting has an equal chance of finding Maxwellian spectra, low kappa value spectra, peaked spectra, spectra from wave‐particle interactions that exhibit localized maxima, electron beamed spectra, or any other shape. By maximizing both in DEF and in energy, the electron spectra, if precipitating, would determine the largest amount of energy that could be deposited in the atmosphere of Mars.

After the initial sort was completed, the top 10 spectra from sectors 0 and 15 were still dominated by the patterns that showed the instrument interference that was linked to patterns of noise. The spectrograms showed that the noise was wide ranging and dominated the high energies above 1 keV, so these two sectors were excluded from use as their values were considered not reliable. The spectra from sectors 12, 13, and 14 were also examined. It was assumed that sectors 12–14 might be dominated by fluxes during time periods when the ASPERA‐3 scanner moved into a position when these sectors pointed into open space; however, the energy spectra from sectors 12–14 showed substantially higher fluxes at low energy (again, these are times of very high flux), which indicated that the selected times were dominated by emission from the spacecraft surface and did not supply an accurate measurement of the environment around Mars. These sectors were also excluded. The resulting spectra were required to be continuous in energy. Spectral data from category 4 (from 5 to 10 keV) were found to be from random points, not forming a continuous spectrum. Thus, category 4 data were excluded from further study. From the remaining data, the criteria of the largest DEF value for energies above the DEF peak showed that spectra that were large in DEF for categories 1 (from ~150 to 500 eV) and 2 (from 500 eV to 1 keV) showed large DEF spectra that did not extend into the 1‐ to 5 keV range. This means that their energy would be deposited at higher vertical altitude or over a shorter horizontal distance than spectra identified as belonging to category 3 (from 1 to 5 keV). Category 3 spectra represent electrons that penetrate deeper into a vertical atmosphere and deposit their energy at lower altitudes or along a longer horizontal path length. Note that category 3 spectra may show a DEF peak at energies lower than those of category 3, but in this case, the spectra is more kappa shaped and the nonthermal high energy tail of the distribution extends the energy deposition range to lower vertical altitudes (see Frahm et al., 1997, for the influence of a noninfinite kappa). Thus, only category 3 (from 1 to 5 keV) was examined further.

Some of class A data (the largest value of DEF) were found to contain energy spikes that were often only one energy channel wide and were not representative of the spectrum in general. For these cases, the desire to have large DEF values did not selectively produce continuous spectra. This was probably due to allowing most noise values through the sorting system and not trapping noise values adequately enough to block their presence. Additionally, a few class A data were dominated by large error values that occurred at low count rates. These showed that the largest value of the spectrum was not representative of the spectral shape and the DEF they represented should not have been included within the sorted maximum spectra. The maximum selected DEF spectra determined by class A selection were highly dominated by statistical fluctuations and were found not to produce representative maximum spectra. For this reason, class A data were eliminated from further study.

From the remaining 110 possibilities, the values were resorted from the highest DEF to the lowest DEF. Only the sector with the largest DEF was chosen to be in the top 10, while the remaining sectors from that same time period were excluded. These times are given in Table 1. Table 1 also includes the MEx orbit number, the value of the average DEF in the 1‐ to 5 keV category, the ELS sector where the highest average value of DEF occurred, an approximation of the peak energy determined visually from the spectra (shown later in Figures 5 and 6), and the estimated isotropic electron energy from the selected spectrum between 1 eV and 5 keV.

**Table 1:** The Top 10 Largest Differential Energy Flux (DEF) Values Recorded at Mars Between 2004 and 2016 Selected From the 1‐ to 5 keV Category of Class B

Top 10 order	Year	Day of year	Date	MEx orbit	Time (UT)	Average DEF in 1–5 keV^a^		Peak energy (eV) b	ELS sector	Electron flux (mW/m^2^)^c^
						erg/(cm^2^ s sr eV)	eV/(cm^2^ s sr eV)			
a	2011	194	13 Jul	9607	00:28:08:941	1.7925 × 10^−4^	1.1189 × 10^8^	~450	06	21.4
b	2005	261	18 Sep	2156	10:22:06.941	1.7916 × 10^−4^	1.1184 × 10^8^	~750	11	29.9
c	2010	067	8 Mar	7921	17:34:31.908	1.3001 × 10^−4^	8.1155 × 10^7^	~1100	09	5.5
d	2005	260	17 Sep	2154	20:52:05.512	8.5435 × 10^−5^	5.3330 × 10^7^	~500	06	15.7
e	2011	157	6 Jun	9482	14:55:23.168	5.3163 × 10^−5^	3.3185 × 10^7^	~350	09	25.0
f	2005	260	17 Sep	2153	14:09:40.028	3.3922 × 10^−5^	2.1175 × 10^7^	~400	05	7.3
g	2010	288	15 Oct	8680	20:27:00.400	3.2117 × 10^−5^	2.0048 × 10^7^	~600	05	12.6
h	2013	067	8 Mar	11681	06:05:27.292	3.1825 × 10^−5^	1.9866 × 10^7^	~130	10	11.3
i	2011	193	12 Jul	9605	10:31:56.740	2.8969 × 10^−5^	1.8083 × 10^7^	~150	06	15.5
j	2005	057	26 Feb	1428	13:31:54.152	2.3332 × 10^−5^	1.4564 × 10^7^	~550	05	2.5

a 1 erg/(cm^2^ s) = 1 mW/m^2^.

b Approximate values visually determined from the spectra shown in Figures 3 and 4.

c Estimated isotropic electron flux from the spectrum selected between 1 and 5 keV.

## 5 Data From Mars

**Figure 2:**
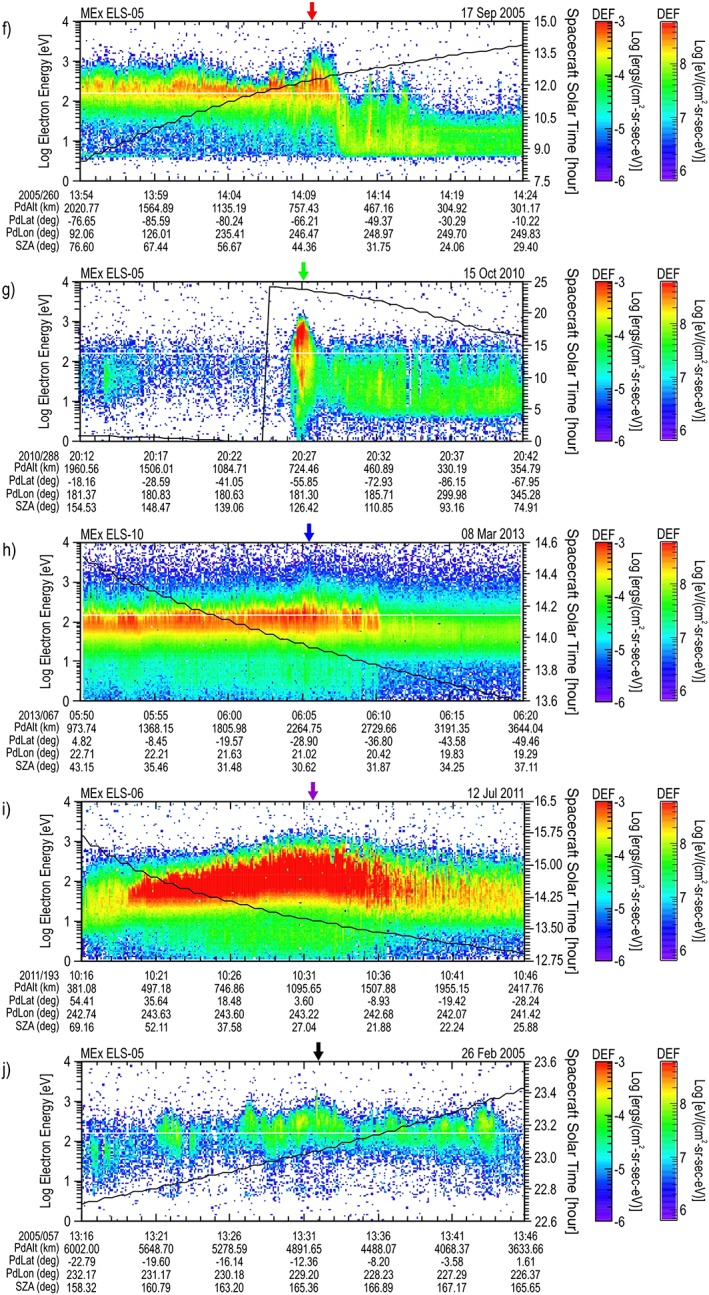
Second five maximum differential energy flux (DEF) energy‐time spectrograms. The order (f–j) is that listed in Table 1. The overlay and bottom plot label definitions are the same as in Figure 1. An arrow at the top of each panel marks the location of the largest DEF spectrum (the color of this arrow corresponds to the color of the orbit presented in Figure 4 and the maximum DEF spectrum presented in Figure 6).

**Figure 3:**
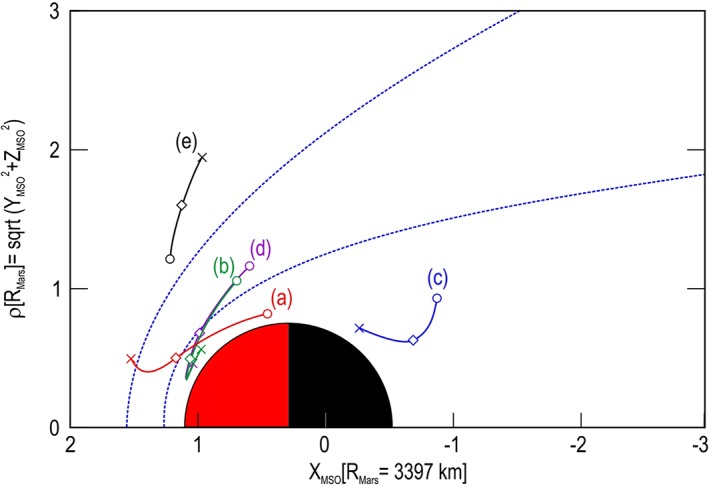
Orbital location of top five. Shown are the orbital locations in cylindrical Mars Solar Orbital (MSO) coordinates from which the top five spectra were collected around Mars. The horizontal axis is the MSO *X* direction, which points toward the Sun (at left). The vertical axis is the cylindrical radius, *ρ*, formed from the perpendicular MSO *Y* and *Z* components. Circles correspond to the beginning times of the spectrograms shown in Figure 1, and crosses, the end times. The event locations are marked with diamonds. The empirical average bow shock (outer boundary) and the magnetic pileup boundary (inner boundary) based on Vignes et al. (2000) are shown (blue conical shaped dashed lines).

**Figure 4:**
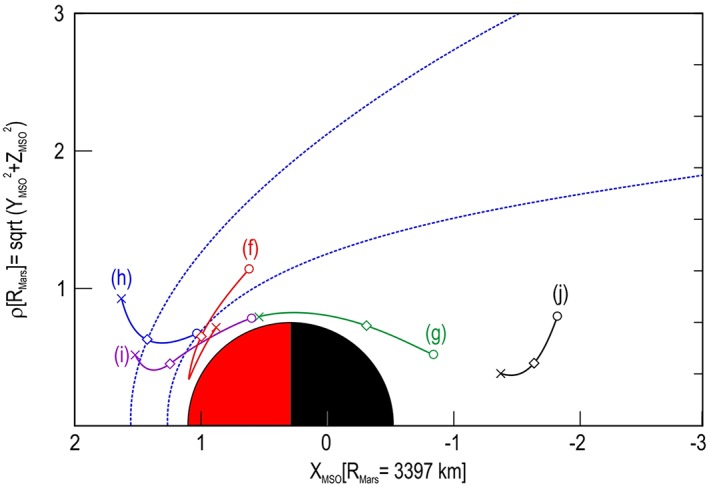
Orbital location of second five. The format is the same as Figure 3, except that shown are the orbital locations for those spectrograms presented in Figure 2.

**Figure 5:**
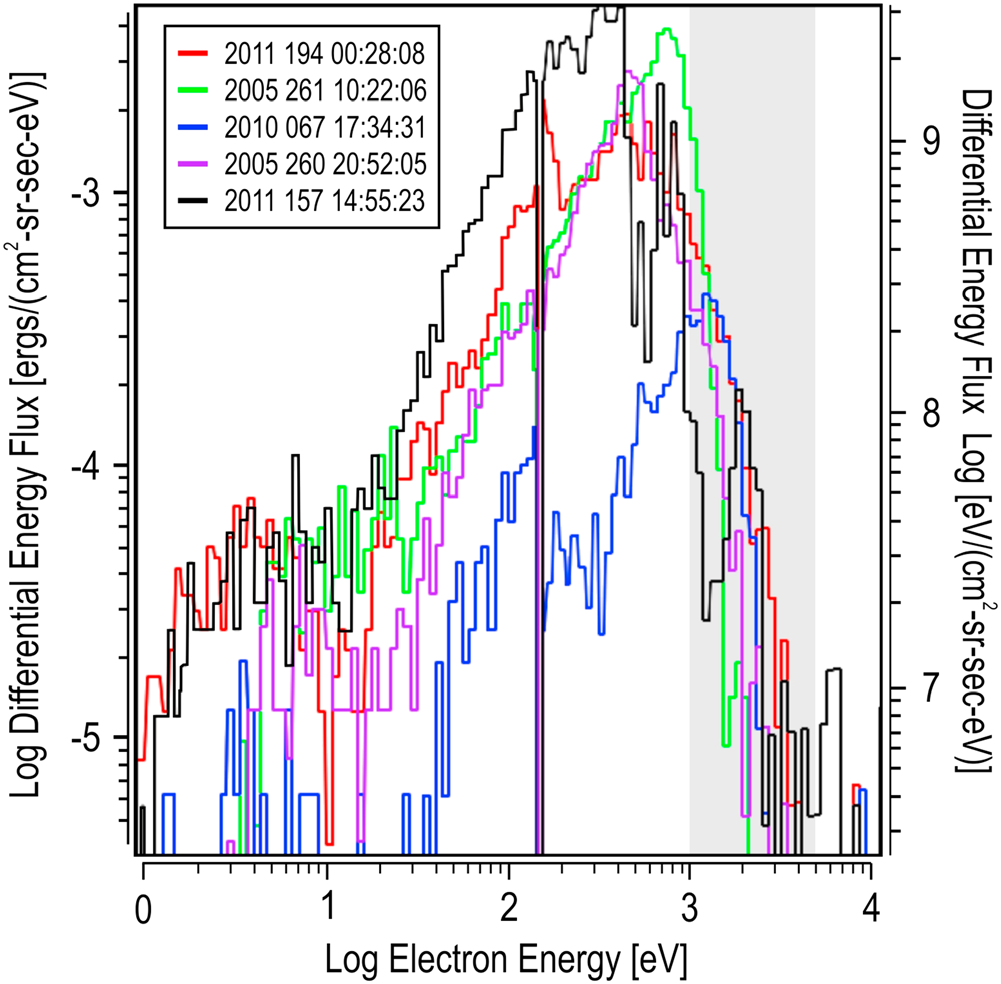
Top five maximum differential energy flux spectra. Each differential energy flux energy spectrum represents a slice of the spectrogram shown in Figure 1 indicated at the location of the arrows and listed as entries (a–e) in Table 1. Each spectrum is color coded, matching the spectrogram's arrow and indicated in the legend.

**Figure 6:**
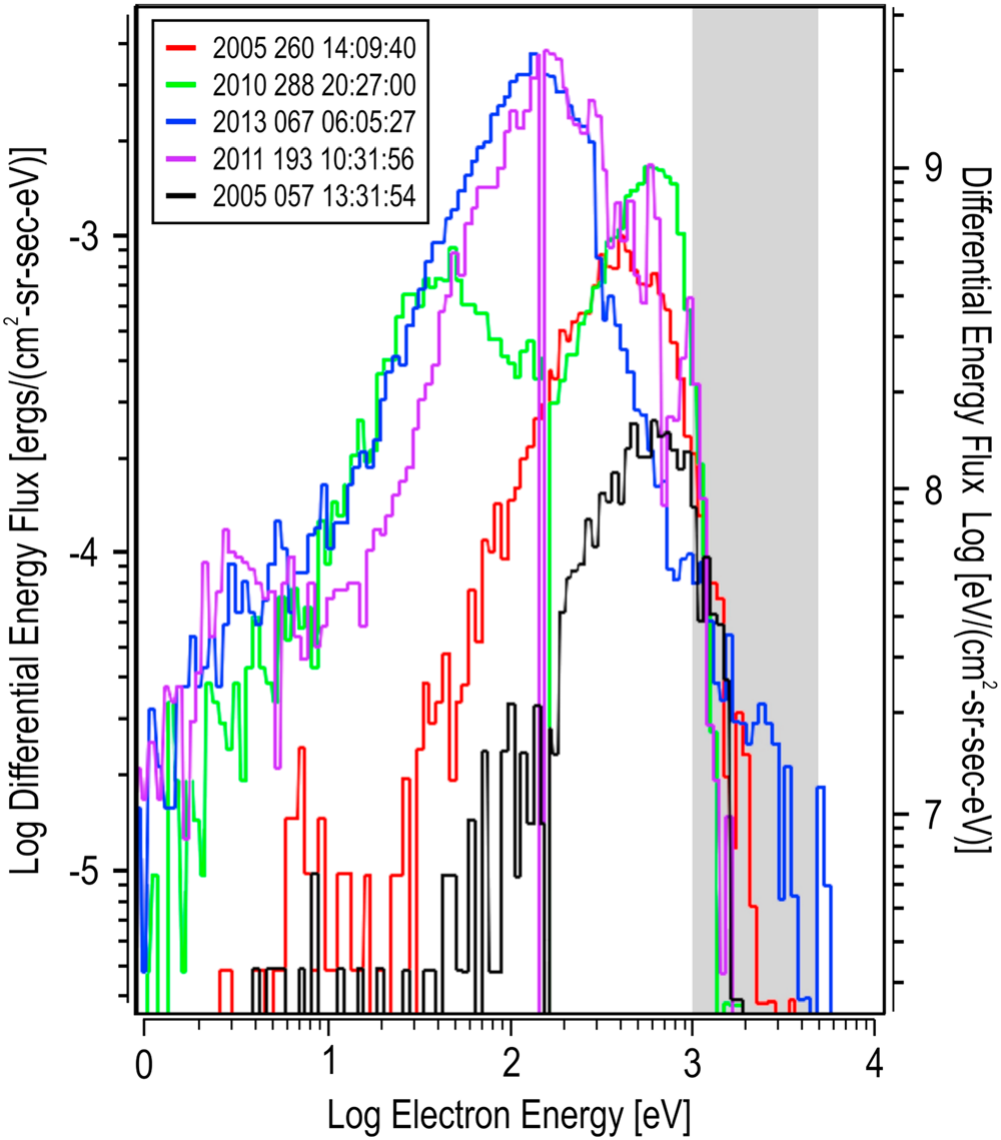
Second five maximum differential energy flux spectra. Each differential energy flux energy spectrum represents a slice of the spectrogram shown in Figure 2 indicated at the location of the arrows and listed as entries (f–j) in Table 1. Each spectrum is color coded, matching the spectrogram's arrow and indicated in the legend.

The spectrograms containing the top five maximum DEF times are presented in Figure 1, and the second five are presented in Figure 2, ordered highest to lowest as shown in Table 1. Each spectrogram is in electron energy‐time format covering 30 min, with the DEF shown using the color bar at the right of the spectrogram (note that the DEF range is selected to be the same for each spectrogram, chosen for ease of comparison). Each spectrogram contains an overlay of the solar local time (hr) with the scale on the right axis. At the bottom of each plot the values of planetodetic (the shape of the planet is approximated by an ellipsoid) altitude (PdAlt in km), planetodetic latitude (PdLat in deg), and planetodetic longitude (PdLon in deg) along with the solar zenith angle (SZA in deg) of the spacecraft are given. An arrow at the top of each panel marks the location of the largest DEF spectrum (the color of this arrow corresponds to the color of the orbit location presented in Figures 3 and 4 and the maximum DEF spectrum presented in Figures 5 and 6).

For the data presented in 2005, the ELS protection screen was set at −5 V, repelling all electrons less than 5 eV. At the time of design, the low‐energy environment on Mars was unknown. Based on experience at the Earth (Burch et al., 1981; Heikkila et al., 1970; Winningham et al., 1981, 1993) and the Mars Global Surveyor results from the Electron Reflectrometer (Acuña et al., 1992), it was anticipated that the thermal population of electrons with energy less than 5 eV could overwhelm the instrument. Thus, taking a cautious approach, a protection grid to block low‐energy electrons from the ELS sensor was installed. Gradually, the protection grid potential was reduced to unveil the lowest energies around Mars. In the data presented from 2010 and beyond, the ELS protection grid was set to ground, allowing low‐energy particles to reach the ELS detector. The actual lowest energy measured is a function of the spacecraft potential. MEx contains no instrument to measure spacecraft potential. In this study, the electron spectrum is not corrected for the potential of the spacecraft. However, the correction for spacecraft potential is estimated to be about the 1% level for 1 keV electrons. This estimation is based on the energy level of the thermal electron signature observed in some spectrograms when the spacecraft is positively charged, accelerating enough thermal electrons into the energy range of ELS.

Two oddities can be observed in the data figures shown. The first can be seen in the spectrogram shown in Figure 1d. The data between 17 September 2005 (260) at 20:41:08 UT and 20:49:35 UT suffered from a thermal instability that was later corrected by rotating ASPERA‐3 communication cables away from direct Sun exposure. The second is the junction between the two ELS power supplies as present across the entire period of Figure 1d. This is observed as a small gap in the energy scan at about 150 eV. In both of these cases, there is no effect on the conclusions drawn by this paper.

It is noted that only three of the top 10 spectra (c, g, j) occur on the nightside of the planet. In all three of these nightside cases, the spacecraft is in the umbra of the planet; however, spectrum g is closer to the edge of darkness, and spectra c and j are encountered deeper in the darkness. The spectra from pass a, b, d, e, f, h, and i occur within a few hours of noon. The maximum spectra on the dayside occur as follows: a—mid‐magnetosheath, b—ionospheric side of the magnetosheath, d—mid‐magnetosheath, e—bow shock side of the magnetosheath, f—ionospheric side of the magnetosheath, h—bow shock side of the magnetosheath, i—mid‐magnetosheath. With the exception of g, spectra in the tail contain lower isotropic energy flux between 1 eV and 5 keV than in the magnetosheath.

The location of the orbits shown in Figure 1 for the top five are presented in Figure 3, and those from the second five shown in Figure 2 are presented in Figure 4. Each figure shows an orbit segment displayed by using the same color as the arrow marking the largest DEF spectrum on the corresponding spectrogram. The plot is displayed using Mars Solar Orbital coordinates (*X* direction points from Mars toward the Sun, *Z* is perpendicular to the planet's velocity vector and is directed toward the northern ecliptic hemisphere, and *Y* completes the right‐handed, orthogonal system) in a cylindrical system with the *X* direction along the axis of the cylinder (toward the Sun) and the *Y* and *Z* axis forming the radius *ρ* (*ρ* = sqrt(*Y* ^2^ + *Z* ^2^)) perpendicular to the Mars‐Sun line. Here the radius on Mars is 3,397 km. For each orbital segment, a circle marks the spacecraft location at the beginning time on the spectrogram, a cross marks its location at the end time shown on the spectrogram, and a diamond marks the location where the large DEF spectrum occurred.

The spectra at the times given in Table 1 were extracted. The first five are shown in Figure 5, and the second five are shown in Figure 6. These figures present the spectra in terms of DEF versus the electron energy. The region of selection, between 1 and 5 keV, is noted with a shaded region on the graphs. The electron energy peak of the DEF spectrum is estimated from these figures and shown in Table 1.

For the top five spectra shown in Figure 5, four dayside spectra and one spectrum on the nightside are represented. All of the dayside spectra have about an order of magnitude larger peak DEF than the nightside pass; however, the nightside case shows energies greater than 1 keV that are comparable to the dayside spectra. Even though the nightside spectrum shows a lower DEF, the peak energy (c: ~1.1 keV, b: ~750 eV, d: ~500 eV, a: ~450 eV, e: ~350 eV) was the highest measured.

For the second five spectra shown in Figure 6, the intensity of the DEF peaks are significantly lower. The estimated energy of the DEF peaks also show lower energies in general (g: ~600 eV, j: ~550 eV, f: ~400 eV, i: ~150 eV, h: ~130 eV). However, above 1 keV, the shapes of all spectra are comparable. A slightly larger, higher energy at lower flux exists for h, which could be caused by a small peak in the DEF at about 2 keV; however, at this low DEF intensity, the count rates have a large uncertainty, and this feature could be purely statistically generated. It is noted that a similar double peak electron energy spectra previously identified in the Martian magnetotail region as presented by Soobiah et al. (2013) is observed in spectrum g.

## 6 Discussion

As discussed in section 2, ELS measures from a plane and relies on a scanner to rotate that measurement plane to sample from the full sky. Because the ELS does not sample continuously from the full sky, it is likely to be sampling the electron distribution at angles other than that which contains the maximum flux. However, it is assumed that within the 24 million spectra sampled, the maximum spectra found will be representative of the largest electron distributions during the peak. Because MEx contains no magnetometer, it is not possible to identify which spectra are precipitating, and without a magnetic field model, it is not possible to track the electron distribution to the planet. In addition, larger energy electrons (which have a larger gyroradius) may be more unmagnetized than those of lower energy and could precipitate toward Mars with a range of pitch angles, so the electron distributions presented may contain a mixture of electrons that are confined to different pitch angle ranges. Again without a magnetometer and a magnetic field model, this cannot be confirmed.

Without preconceived notion of the electron spectrum, we have examined all of the MEx ELS DEF electron energy spectra from the beginning of 2004 to the end of 2016. Of the approximately 24 million spectra sampled over 13 years in the environment of Mars, MEx has sampled electrons from every region and almost every plasma condition. All of these spectra were examined to find the continuous spectrum having the largest DEF at energies above the DEF peak. From this search the largest top 10 spectra were retained. Of the top 10 spectra, seven were found to be from the magnetosheath and only three were found to be from the tail. These three tail spectra exhibit high fluxes at high energy that are not larger than spectra in the magnetosheath, and the high‐energy shape of the spectrum (between 1 and 5 keV in energy) was similar to those from the magnetosheath. Since MEx reaches altitudes where auroral acceleration processes could energize electrons, one would expect that ELS has surveyed spectra that would show an accelerated population if one existed, which could be larger in energy than the magnetosheath spectrum. The fact that the tail spectra are no larger in energy than spectra in the magnetosheath suggests that the planet has no means to accelerate electrons to energies above that which is generated by the interaction of the planet with the solar wind. The fact that the high‐energy population between the magnetosheath spectra and tail spectra are similar suggests that the electrons from the magnetosheath could be redirected (or channeled) by the magnetic field from the magnetosheath to the Martian tail, such that the high‐energy portion of the energy spectra would not be affected. This would result in similar high‐energy tail spectra compared to those measured in the Martian tail.

The top 10 spectrograms indicate that largest spectra are observed in the magnetosheath near the bow shock, in the mid‐magnetosheath, and toward the ionospheric side of the magnetosheath. This suggests that the shape of the electron spectrum is determined at the bow shock and the signature of these high‐energy electrons may be transmitted through the magnetosheath from the bow shock to the ionosphere. It is possible that the high‐energy electrons could skirt along the magnetosheath/ionosphere boundary and precipitate in the tail. The fact that the high‐energy population is observed in the noon‐1400 hr local time sector most likely has to do with the energy imparted to the compressed plasma, and it may exhibit reduced energy from rarefraction as the population travels into the nightside, and then compression again increases the energy to similar levels as the original spectra.

Speculating on more possibilities, it may be that the dayside compression mechanism is somehow duplicated in the tail region of the planet. If the solar wind interplanetary magnetic field (IMF) interconnects with the remnant Martian magnetic field as suggested by Brain et al. (2003), a similar amount of magnetic energy may be imparted to the electrons when closed field lines reform. The electron signatures may also be related to the nightside strip electrons reported by Dubinin et al. (2006) since oscillations observed in magnetosheath electrons are also observed in strip electrons, even though narrow beams of planetary ions are observed at the same time as strip electrons, suggesting connection to the ionosphere.

Note that no spectrum came from the magnetosheath in the flanks of Mars where the plasma is accelerating around the planet, nor were there any from around the polar region where the stretched magnetic field would be slipping around the planet. No spectrum came from the dayside ionosphere where compression might be the largest or in the ionosphere from the terminator region where the ionization sources from the dayside dominate and the ionization sinks from the nightside dominate. The lowest‐altitude spectrum occurs on the nightside at about 750 km altitude despite ELS making measurements to altitudes of 250–350 km. No spectrum was identified in the lower‐altitude regions in the southern hemisphere where the crustal magnetic fields are expected to accelerate plasma. The dominant DEF spectra at large energy come from the dayside magnetosheath of the planet or the nightside umbral tail.

Discrete Martian “aurora” has been detected on the nightside of Mars. The Spectroscopy for the Investigation of the Characteristics of the Atmosphere of Mars (SPICAM; Bertaux et al., 2004) has identified optical signatures that were attributed to “auroral” electrons. SPICAM infers the electron spectrum based on the line‐of‐sight optical signature. At the time period of the intensification in Figure 2g, the SPICAM instrument has identified an optical signature that they have defined as an auroral signature. This feature was also discussed in Gérard et al. (2015). Their measured ultraviolet (UV) intensity was the strongest reported in the CO_2_^+^ UV doublet (which is the most direct proxy of the auroral energy flux interacting with the atmosphere) at 288.3 and 289.6 nm while SPICAM was pointing in the nadir direction. This feature was reported to exhibit both the highest value of the electron energy flux (reaching up to 10.2 mW/m^2^) and the highest electron energy peak (530 eV) among their time periods when UV was detected. The SPICAM peak values of the UV emission and the ASPERA‐3 electron energy flux were separated by about 10° in latitude. Note that for the electron flux estimated for the largest DEF, the estimated isotropic electron flux is slightly higher at 12.6 mW/m^2^ (Table 1).

Since MEx does not carry a magnetometer, it is not possible to uniquely map the magnetic field between the location of in situ electron measurement at the spacecraft to the location where the peak UV signature was generated in the atmosphere of Mars, meaning it is not possible to show uniquely that the same electrons that are measured in situ by ELS are the same electrons that produce the optical signature remotely observed by SPICAM. No current magnetic field model taking into account both internal and external magnetic fields exists for this mapping purpose. However, close occurrence between the times UV signature measured in the nadir direction and in situ electron measurements has been argued (Gérard et al., 2015; Leblanc et al., 2008; Soret et al., 2016).

**Figure 7:**
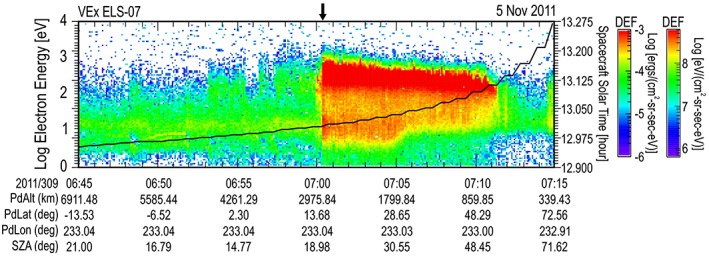
The spectrogram containing the largest differential energy flux (DEF) measured at Venus between 2006 and 2014. The format is similar to that presented in Figure 1. The arrow at the top of the spectrogram indicates the location of the maximum spectrum.

**Figure 8:**
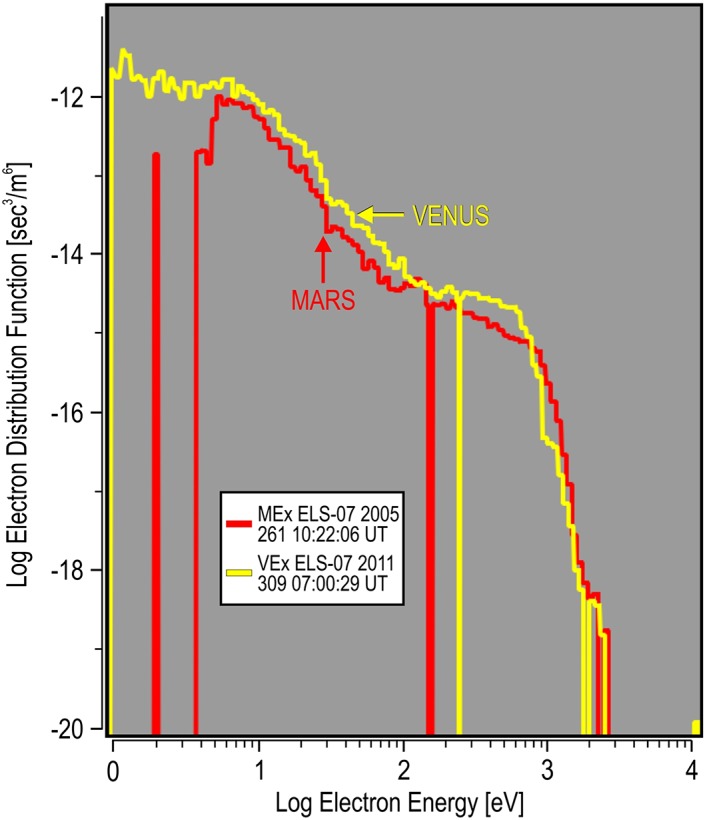
The largest Venus differential energy flux energy spectrum compared to the large Mars differential energy flux. The spectrum at Mars is taken from the inner magnetosheath shown in Figure 1b and Table 1. Comparison between planets is shown in units of the electron distribution function.

**Figure 9:**
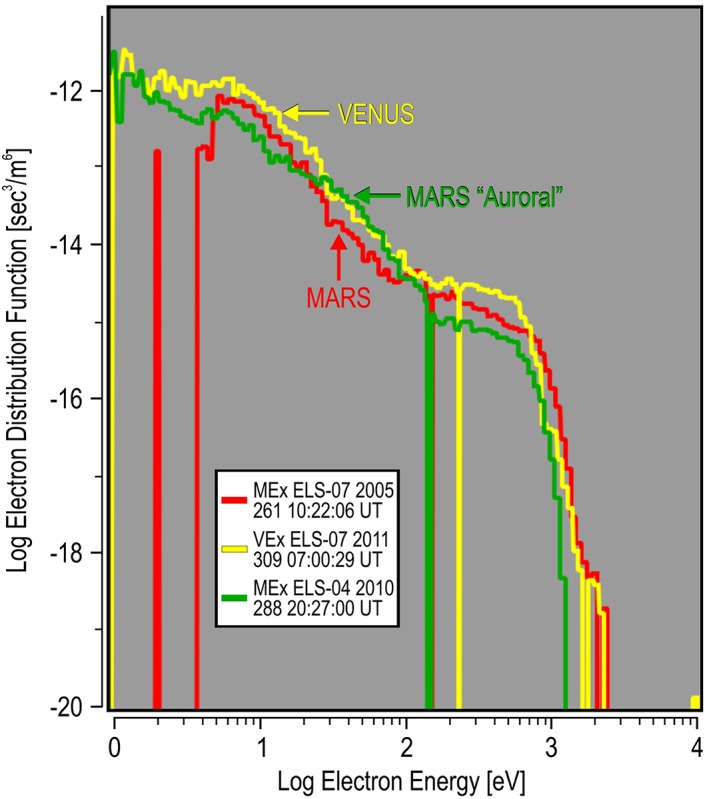
The largest Venus differential energy flux energy spectrum compared to the large Mars differential energy flux spectrum from the inner magnetosheath (Figure 1b) and the “auroral” spectrum (Figure 2g). Format is similar to Figure 8.

In order to find out if the large electron spectra are uniquely related to the magnetic field of Mars, a similar search was conducted for the largest DEF spectra observed at Venus between 2006 and 2014 using the similar ELS from the ASPERA‐4 experiment (Barabash et al., 2007) of the Venus Express (VEx) spacecraft. Venus has no internal magnetic field. Its magnetic deflection is totally induced. The largest value of DEF was found to occur on 5 November 2011 (309) at 07:00:29 UT and listed in Table 2. The ASPERA‐4 ELS Venus spectrogram for sector 07 is shown in Figure 7. This largest value was observed in the magnetosheath of Venus near the bow shock, post noon. The spectrum from the Venus bow shock is compared to the highest DEF spectrum at the inner edge of the Mars magnetosheath (b) and is shown in Figure 8. Comparison was made between the energy spectrum of the DF (s^3^/m^6^) from both planets. Both spectra were found to be similar, with the spectrum from Venus being of slightly larger magnitude below 1 keV and the Mars spectrum being slightly larger above 1 keV. Without knowing the planet from which the spectrum came, it would not be possible to tell the difference. In fact, an additional comparison with the nightside auroral spectrum (g) shows a similar distribution to both the Mars and Venus spectra from the magnetosheath in front of the planets (Figure 9). Again, the shapes are very similar, and it is hard to tell the difference between all three spectra. Here the Mars auroral spectrum is the least intense above 100 eV and falling faster in DF above 1 keV when compared to the Mars and Venus magnetosheath spectra. In the auroral spectrum, there is no clear peak in the DF between 200 eV and 1 keV, indicating that there was no additional acceleration process required to generate this spectrum. For electrons below 200 eV, there is not enough information presented to determine if there is a further potential drop that influences the low‐energy electrons. An additional observation is that the Mars auroral spectrum hints at being from a larger kappa population than the magnetosheath spectra, meaning that it has a more thermalized high‐energy tail and a more Maxwellian shape. This would translate to energy deposition at higher altitudes during precipitation than the magnetosheath spectrum (again, assuming precipitation).

**Table 2:** Comparison Spectra From Venus Between 2006 and 2014, and Earth From 1981

Planet	Year	Day of year	Date	Time (UT)	Average DEF in 1–5 keV^a^		Peak energy (eV)^b^	Sector	Electron flux (mW/m^2^)^c^
					erg/(cm^2^ s sr eV)	eV/(cm^2^ s sr eV)			
Venus	2011	309	5 Nov	07:00:28.923	4.6365× 10^−5^	2.8942× 10^7^	580	ELS‐07	33.8
Earth	1981	296	23 Oct	04:46:31.232	2.3080× 10^−5^	1.4407× 10^7^	600	LAPI‐7.5	10.1

a 1 erg/(cm^2^ s) = 1 mW/m^2^.

b Approximate values visually determined from the spectra.

c Estimated isotropic electron flux.

**Figure 10:**
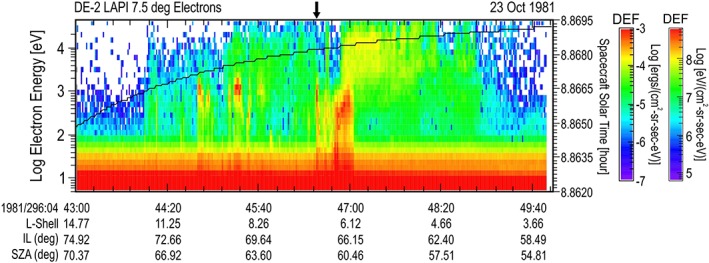
An auroral zone pass from the Earth observed by the Low Altitude Plasma Instrument flown on the Dynamics Explorer‐2 satellite. Format is similar to the spectrograms shown in Figure 1. Parameters listed at the bottom of the spectrogram are the L‐shell, invariant latitude (IL), and solar zenith angle (SZA) of the satellite.

**Figure 11:**
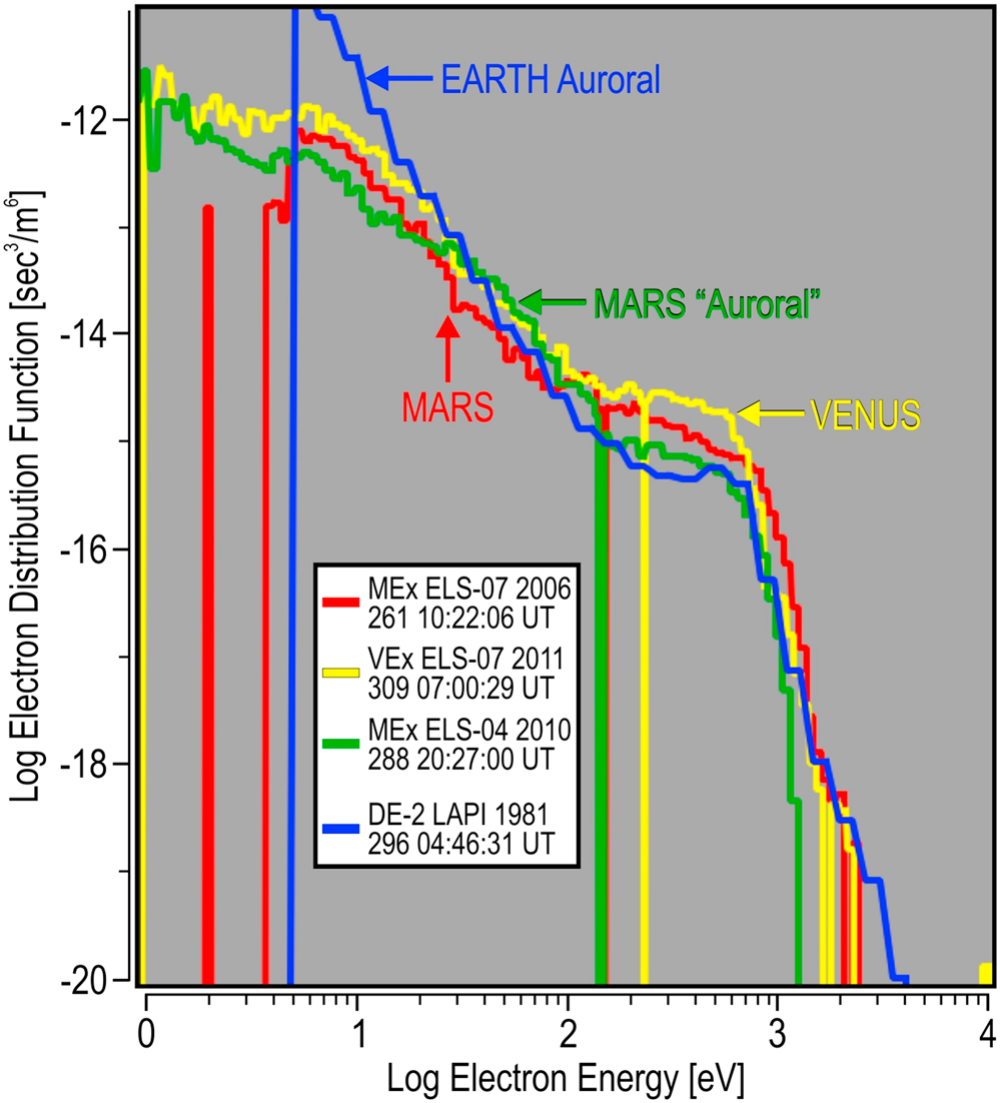
The largest differential energy flux energy spectrum at Venus, a dayside inner magnetosheath spectrum at Mars, the nightside “auroral” spectrum at Mars, and an auroral spectrum from the Earth are shown. Comparison between planets is shown in units of the electron distribution function.

Auroral spectra have been observed at Earth by many spacecraft as they pass through the auroral zone. Shown in Figure 10 are data from the 7.5° sensor on the Low Altitude Plasma Instrument (LAPI; Winningham et al., 1981) flown on the Dynamics Explorer‐2 (DE‐2) satellite. DE‐2 orbited Earth with an approximately polar trajectory through the auroral zone at about 350 km altitude. Typical auroral zone pass contains spectra that have DEF peaks in the 1 to 10 keV energy range. A less energetic spectrum with similar flux in the 1 to 5 keV energy range was chosen from a single randomly selected satellite pass through the auroral zone. This pass occurred on 23 October 1981 (296) at 04:46:31 UT (also listed in Table 2) and is shown in Figure 10. This Earth spectrum was compared to the DFs observed at Mars and Venus, shown in Figure 11. Between energies of 100 eV and 1 keV, the auroral signature at the Earth is nearly identical to the auroral signature at Mars. Differences in the spectra are above 1 keV, suggested by the shape to be different values of a Kappa electron distribution where the larger value of Kappa exists at Mars than at Earth (meaning that the Mars plasma is thermalized to a higher degree). Differences can also be seen below 50 eV where the thermal electron plasma at the Earth is larger. When the auroral spectrum from the Earth is compared to the magnetosheath spectra at Mars and Venus, the shapes are similar above 50 eV. Again, given the spectra without labels, it would be difficult to tell from where they came. When the Earth auroral zone spectrum is compared to the Mars auroral spectrum, there exists a slight decrease in the Earth auroral zone spectrum between 200 eV and 1 keV, which hints that an acceleration mechanism has been applied to accelerate the Earth auroral spectrum.

The spectrogram in Figure 10 also implies that there are many auroral zone spectra at the Earth that are more energetic than at Mars. Although collected decades apart in time, it is clear that the intrinsic magnetic field condition at the Earth allows acceleration mechanisms to exist, which impart energy to the electrons exceeding that which can be imparted to the electrons by the induced interaction of the solar wind at Mars and Venus. The Mars data also imply that there are no additional processes required to generate the “accelerated” electron spectrum in the Martian tail than what is available from the interaction of the planet with the solar wind.

## 7 Conclusion

In order to find electron spectra that limit the possible amount of energy deposited by electrons into the atmosphere of Mars, the ELS instrument was used without any preconceived notion of the shape or location of the electron spectrum. Time periods for the 10 largest DEF at energies above the DEF peak that produced continuous spectra were found from a solar cycle of data. By selection of energy spectra in the 1 to 5 keV energy range, these 10 cases should have the greatest influence on the atmosphere around the atmospheric energy deposition peak if the electrons vertically precipitated. The 10 largest time periods were required to be from different orbits. The observations covered a solar cycle from January of 2004 to December of 2016. For each time period, only the sensor with the largest observed DEF was selected. This produced seven spectra from the dayside, within 2 hr just after noon, and three spectra from the nightside where the spacecraft was in the shadow of Mars. The dayside spectra came from the magnetosheath: near the bow shock, in the mid‐magnetosheath, and near the ionosphere.

The top 10 spectra all showed a very similar shape in their DEF‐electron energy profile at energies from slightly greater than the peak DEF until the instrument threshold, which most likely reflects a limit to the amount of energy that could be imparted to the electrons. Electron spectra from the dayside reflect the impact of the solar wind on the induced magnetosphere of Mars. Electron spectra observed from the tail achieved this similar limiting energy, suggesting that the planet imparts no more energy to the electrons than is provided by the solar wind‐planet interaction. Rarely is a peak DEF larger than 1 keV found at Mars. This is unlike Earth, where spectra in the auroral zone vary substantially with peak DEF energies that can exceed above 10 keV.

A similar study at Venus using the ELS instrument of the VEx ASPERA‐4 experiment found that the maximum continuous DEF spectrum with the maximum energy occurred near the Venus bow shock, just inside the magnetosheath. When the Venus spectrum from near the bow shock was compared to a Mars spectrum near the ionosphere in the magnetosheath, they exhibited the same shape to the point that without prior knowledge, they could not be distinguished from each other. When compared to a nightside auroral electron spectrum at Mars, the shapes of all three spectra were found to be very similar.

A comparison of spectra was made to one from the auroral zone of Earth. The Earth spectrum was chosen to have a similar maximum energy as that from the induced magnetospheres of Mars and Venus. This spectrum was similar in shape but did exhibit more of a deviation than the spectra from the planets with the induced magnetospheres. However, additional spectra from the Earth auroral zone showed that the higher energy extent reached much greater maximum energies than at Mars or Venus, indicating that the intrinsic field of the Earth is capable of imparting much more energy into the electron spectrum than what is available in the interaction of the solar wind with induced magnetic field planets.
